# Temporal Trends and Machine Learning-Based Risk Prediction of Female Infertility: A Cross-Cohort Analysis Using NHANES Data (2015–2023)

**DOI:** 10.3390/diagnostics15172250

**Published:** 2025-09-05

**Authors:** Ismat Ara Begum, Deepak Ghimire, A. S. M. Sanwar Hosen

**Affiliations:** 1 Department of Biomedical Sciences and Institute for Medical Science, Jeonbuk National University Medical School, Jeonju 54907, Republic of Korea; ismatara1986@gmail.com; 2 IT Application Research Center, Korea Electronics Technology Institute, Jeonju 54853, Republic of Korea; 3 Department of Artificial Intelligence and Big Data, Woosong University, Daejeon 34606, Republic of Korea

**Keywords:** female infertility, NHANES, machine learning, logistic regression, risk prediction, reproductive health, ROC curve

## Abstract

**Background:** Female infertility represents a significant global public health concern, yet its evolving trends and data-driven risk prediction remain under examined in nationally representative cohorts. This study investigates temporal changes in infertility prevalence and evaluates Machine Learning (ML) models for infertility risk prediction using harmonized clinical features from NHANES cycles (2015, 2016, 2017, 2018, 2021, 2022, and 2023). **Methods:** Women aged 19 to 45 with complete data on infertility-related variables (including reproductive history, menstrual irregularity, Pelvic Infection Disease (PID), hysterectomy, and bilateral oophorectomy) were analyzed. Descriptive statistics and cohort comparisons employed ANOVA and Chi-square tests, while multivariate Logistic Regression (LR) estimated Adjusted Odds Ratios (OR) and informed feature importance. Predictive models (LR, Random Forest, XGBoost, Naive Bayes, SVM, and a Stacking Classifier ensemble) were trained and tuned via GridSearchCV with five-fold cross-validation. Model performance was evaluated using accuracy, precision, recall, F1-score, specificity, and AUC-ROC. **Results:** We observed a notable increase in infertility prevalence from 14.8% in 2017–2018 to 27.8% in 2021–2023, suggesting potential post-pandemic impacts on reproductive health. In multivariate analysis, prior childbirth emerged as the strongest protective factor (Adjusted OR ≈0.00), while menstrual irregularity showed a significant positive association with infertility (OR =0.55, 95% CI 0.40 to 0.77, p<0.001). Unexpectedly, PID, hysterectomy, and bilateral oophorectomy were not significantly associated with infertility after adjustment (p>0.05), which may partly reflect the inherent definition of self-reported infertility used in this study. All six ML models demonstrated excellent and comparable predictive ability (AUC >0.96), reinforcing the effectiveness of even a minimal common predictor set for infertility risk stratification. **Conclusions:** The rising prevalence of self-reported infertility among U.S. women underscores emerging public health challenges. Despite relying on a streamlined feature set, interpretable and ensemble ML models successfully predicted infertility risk, showcasing their potential applicability in broader surveillance and personalized care strategies. Future models should integrate additional sociodemographic and behavioral factors to enhance precision and support tailored interventions.

## 1. Introduction

Infertility, defined as the inability to achieve pregnancy after 12 months of regular unprotected intercourse, affects an estimated 10–15% of reproductive-aged couples globally, representing a significant public health challenge with psychological, social, and economic consequences [1,2,3,4]. In women, infertility arises from a complex interplay of factors including ovulatory dysfunction, tubal obstruction, endometriosis, pelvic infections, uterine abnormalities, and age-related ovarian decline [5]. While male factors also contribute, female reproductive health has received particular attention due to its multifaceted and often under-recognized nature in clinical settings.

In recent years, shifts in lifestyle, environmental exposures, delayed childbearing, and increased prevalence of conditions like Polycystic Ovary Syndrome (PCOS) and obesity have raised concern about a potential rise in infertility rates across high-income countries [6,7]. The COVID-19 pandemic has further amplified these concerns, as it disrupted access to reproductive healthcare services, delayed fertility treatments, and exacerbated stress-related reproductive dysfunction [8,9,10]. Understanding whether these systemic changes have translated into measurable changes in infertility prevalence remains an urgent research priority.

Nationally representative health surveys such as the National Health and Nutrition Examination Survey (NHANES) offer a valuable resource to investigate infertility trends over time and explore associated risk factors in large, diverse populations. Prior studies utilizing NHANES data have identified reproductive history, menstrual irregularity, Pelvic Inflammatory Disease (PID), and surgical interventions such as hysterectomy or oophorectomy as influential variables in infertility outcomes [11,12]. While such variables, particularly surgical procedures like hysterectomy, may not serve as modifiable predictors, they can reflect the broader reproductive history or endpoints that influence survey-reported infertility and remain useful for population-level risk stratification.

In parallel, advancements in Machine Learning (ML) have opened new avenues for risk stratification in reproductive medicine. ML algorithms such as Random Forests (RF), Naive Bayes (NB), Support Vector Machine (SVM), Extreme Gradient Boosting (XGBoost) and an advanced ensemble method with Stacking Classifier can capture complex nonlinear relationships and variable interactions, offering improved predictive accuracy over traditional statistical models [13,14,15,16]. Despite their potential, these techniques remain underutilized in population-level infertility research, where explainable and scalable tools are needed to inform early identification and intervention.

To our knowledge, this is the first study to combine temporal trend analysis with ML-based risk prediction of female infertility using nationally representative NHANES data spanning both pre- and post-COVID-19 eras (2015–2023) [17]. While prior studies have explored infertility risk factors in earlier NHANES cycles, they have not addressed recent changes in prevalence or applied predictive modeling frameworks. Our study is novel in its dual focus on (1) detecting emerging trends in infertility rates after the pandemic and (2) evaluating interpretable and scalable ML models, such as LR, RF, XGBoost, NB, SVM, and ensemble method, for infertility prediction using a harmonized feature set. This approach provides not only epidemiological insight but also practical tools for population-level screening and early intervention.

The remainder of this article is organized as follows: Section 2 presents the study design, data sources, variable definitions, and statistical and ML methods used for analysis. Section 3 reports the results, including descriptive statistics, temporal trends, and predictive model performance. Section 4 provides a discussion that situates the findings within the broader literature. Finally, Section 5 summarizes the key implications for clinical practice and public health and highlights directions for future research.

## 2. Methods

### 2.1. Data Source and Study Population

This study utilized publicly available data from the NHANES spanning three survey cycles: 2015–2016, 2017–2018, and 2021–2023 [17]. NHANES is a cross-sectional survey conducted by the Centers for Disease Control and Prevention (CDC) to assess the health and nutritional status of the U.S. population through interviews, physical examinations, and laboratory tests. The survey employs a complex, multistage probability sampling design to produce nationally representative estimates.

**Figure 1:**
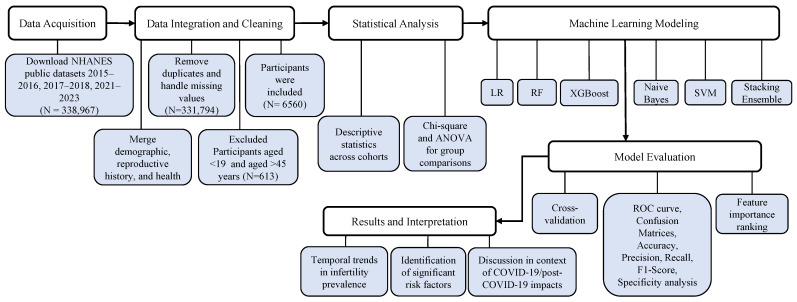
Workflow of data acquisition, processing, statistical analysis, and Machine Learning (ML)-based risk prediction of female infertility using NHANES data (2015–2023).

We included women aged 19–45 years who had complete information on infertility-related variables that were consistently available across all three selected cycles. To ensure comparability, only variables present in all cycles were retained during data harmonization. As shown in Figure 1, a total of 338,967 participants were initially identified across the combined cycles. Of these, 331,794 participants were excluded due to missing, refused, or “don’t know” responses in key reproductive health variables. Subsequently, we excluded 31 individuals who were under 19 years of age and 582 who were over 45 years of age. After all exclusions, the final analytic sample consisted of 6560 women aged 19–45 years who met the inclusion criteria and had complete data for the study variables.

### 2.2. Definition of Infertility

Infertility was defined based on self-reported responses to the reproductive health questionnaire, specifically to the item: “Have you ever attempted to become pregnant over a period of at least a year without becoming pregnant?” Respondents answering “Yes” were classified as infertile (infertile = 1), and others as not infertile (infertile = 0). This question aligns with standard epidemiological definitions of infertility used in population health studies.

### 2.3. Variable Selection and Harmonization

Given the inconsistent availability of several demographic and behavioral variables across NHANES cycles, this study focused on a harmonized subset of clinical and reproductive health variables available in all three cycles. The following predictors were included: Age at Menarche (continuous), Total Deliveries (continuous), Pelvic Infection (binary), Menstrual Irregularity (binary), History of Hysterectomy (binary), Both Ovaries Removed (binary), Ever Pregnant (binary).

Variables such as age, BMI, smoking status, and socioeconomic indicators were excluded from this analysis because they were not available across all three NHANES cycles. While variables like hysterectomy and oophorectomy reflect irreversible reproductive endpoints rather than modifiable risk factors, they were retained in the model to account for variation in reproductive history and its potential influence on self-reported infertility status.

### 2.4. Statistical and Machine Learning Analysis

Descriptive statistics were computed for the overall study population and stratified by NHANES survey cycles (2015–2016, 2017–2018, and 2021–2023). Continuous variables were summarized as means with Standard Deviations (SD), and categorical variables were presented as frequencies with percentages. Group differences across survey cycles were assessed using one-way analysis of variance (ANOVA) for continuous variables and the Chi-square test of independence for categorical variables. Corresponding *p*-values were calculated to evaluate the statistical significance of between-group differences reported in Table 1. All statistical tests were two-tailed, and statistical significance was set at p<0.05.

For inferential analysis, a multivariate LR model was developed to identify independent predictors of infertility, adjusting for potential confounders. Odds Ratios (ORs) and 95% Confidence Intervals (CIs) were estimated, and variables with p<0.05 were considered statistically significant. To further explore predictive modeling, multiple ML algorithms were applied, including LR, RF, XGBoost, NB, SVM, and an ensemble Stacking Classifier. The Stacking Classifier used XGBoost, RF, LR, and SVM as base learners, and LR as the meta-learner. Hyperparameters for each model were optimized using GridSearchCV with five-fold cross-validation. Model performance was evaluated using accuracy, precision, recall, F1-score, and the Area Under the Receiver Operating Characteristic Curve (AUC-ROC). The use of Stacking Classifier in reproductive health prediction has been shown to enhance model robustness, particularly when combined with oversampling strategies such as SMOTE, as demonstrated in recent PCOS prediction research [18]. All statistical analyses were conducted in Python (version 3.9) using the pandas, numpy, scipy, statsmodels, and scikit-learn libraries.

### 2.5. Ethical Considerations

This study used publicly available, identified data from NHANES and therefore did not require ethical approval.

## 3. Results

### 3.1. Temporal Trends in Female Infertility Based on NHANES Data (2015–2023)

**Figure 2:**
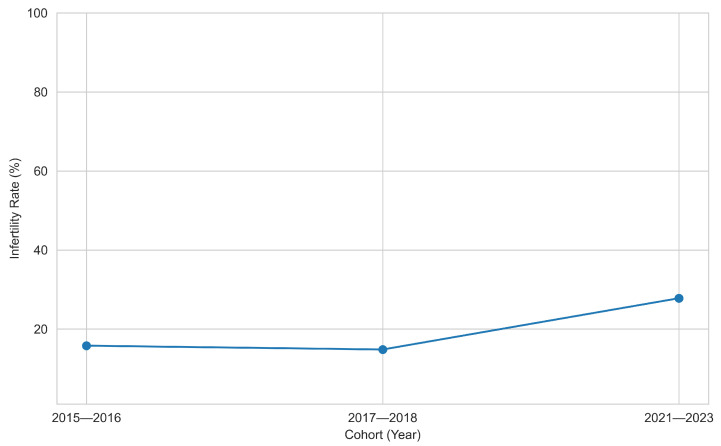
Temporal trends in female infertility.

The proportion of women reporting infertility across three NHANES survey cycles demonstrated a notable shift. In 2015–2016, infertility prevalence was 15.8%, followed by 14.8% in 2017–2018, indicating stability over the pre-pandemic period as shown in Figure 2. However, in the 2021–2023 cycle, prevalence surged to 27.8%, nearly doubling from the previous cycle. This marked increase may be attributed to pandemic-related disruptions, including delayed healthcare access, heightened stress, or changes in reproductive planning. Further investigation is warranted to explore contributing factors to this upward trend.

### 3.2. Descriptive Statistics of the Study Population Based on Common Variables Across NHANES Cohorts (2015–2023)

Table 1 presents the descriptive statistics of the study population based on common variables across NHANES cycles from 2015 to 2023, including a total of 6560 women. The sample sizes for each cycle were 2534 (2015–2016), 2483 (2017–2018), and 1543 (2021–2023).

The mean age at menarche was consistent between the 2015–2016 and 2017–2018 cohorts (both 12.7 ± 1.8 years), but was significantly lower in the 2021–2023 cohort (12.3 ± 1.7 years; p<0.001), yielding an overall mean of 12.6 ± 1.8 years. The mean number of total deliveries was similar in the first two cohorts (2.4 deliveries), but decreased markedly in 2021–2023 (1.5 ± 1.4 deliveries; p<0.001), resulting in an overall mean of 2.2 ± 2.9 deliveries.

The prevalence of menstrual irregularity was highest in the 2021–2023 cohort (65.9%) compared to 46.1% in 2015–2016 and 42.9% in 2017–2018. However, this difference was not statistically significant (p=1.000). Hysterectomy prevalence remained stable in the first two cycles (22.0% and 22.4%), but declined to 11.7% in the most recent cycle; this trend was not statistically significant (p=0.348).

PID was relatively uncommon, ranging from 3.3% in 2015–2016 to 4.9% in 2021–2023, but the variation across cycles was statistically significant (p<0.001). The proportion of women who had ever been pregnant was high in the first two cohorts (84.2% and 85.2%), but decreased substantially in 2021–2023 (72.2%), representing a statistically significant decline (p<0.001).

The percentage of women who had both ovaries removed was stable at approximately 11.3% in the earlier cohorts, but fell to 4.3% in 2021–2023; this reduction approached statistical significance (p=0.070).

Overall, these findings reveal significant changes in several reproductive health indicators over time, particularly in the post-pandemic period (2021–2023). The observed shifts, most notably in mean deliveries, ever-pregnant status, and PID prevalence, may reflect evolving demographic profiles, differences in healthcare utilization, and possible post-COVID-19 influences on reproductive health patterns.

### 3.3. Relative Importance of Common Predictors Across NHANES Cohorts Based on Logistic Regression Coefficients

**Figure 3:**
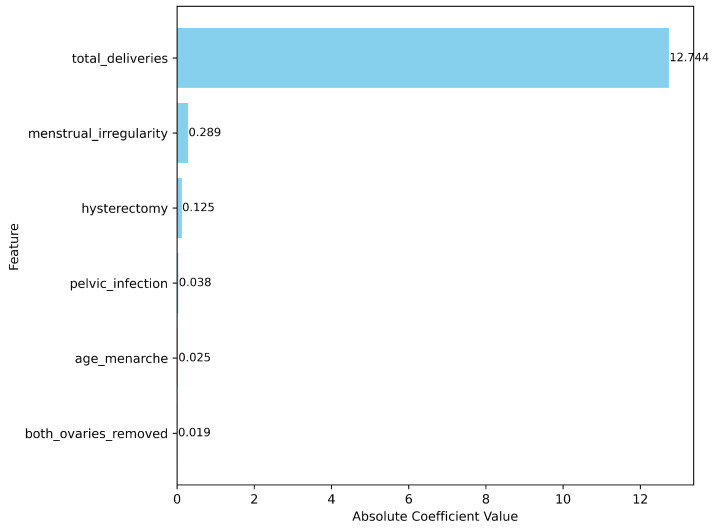
Feature importance of common predictors across NHANES cohorts based on Logistic Regression (LR) coefficients.

To further elucidate the contributions of individual clinical and reproductive history variables to infertility classification, we evaluated the relative importance of common predictors using LR coefficients. Figure 3 illustrates the absolute magnitude of these coefficients, providing an overview of the most influential features in distinguishing women at risk for infertility across the combined NHANES cohorts (2015–2023). Among the six examined variables, total number of deliveries exhibited the most dominant influence, with a markedly higher absolute coefficient (12.744) compared to other predictors. This finding suggests a strong inverse or complex relationship between prior childbirth history and reported infertility, potentially indicating confounding or reverse causality that warrants further investigation.

Following total deliveries, menstrual irregularity showed the next highest coefficient (0.289), reinforcing its established role as a critical clinical indicator of reproductive dysfunction. This was followed by a history of hysterectomy (0.125), a known surgical factor directly impairing fertility. Other variables, including pelvic infection (0.038), age at menarche (0.025), and bilateral oophorectomy (both ovaries removed) (0.019), displayed relatively low coefficient magnitudes, suggesting more modest contributions to infertility classification in this multivariate model.

Overall, these results highlight the disproportionate influence of certain reproductive history factors, especially total deliveries and menstrual irregularity, on infertility classification. The findings underscore the importance of considering both clinical symptoms and life history events in infertility risk assessments and public health strategies.

### 3.4. Infertility Rate by Risk Factor Across Cohorts

**Figure 4:**
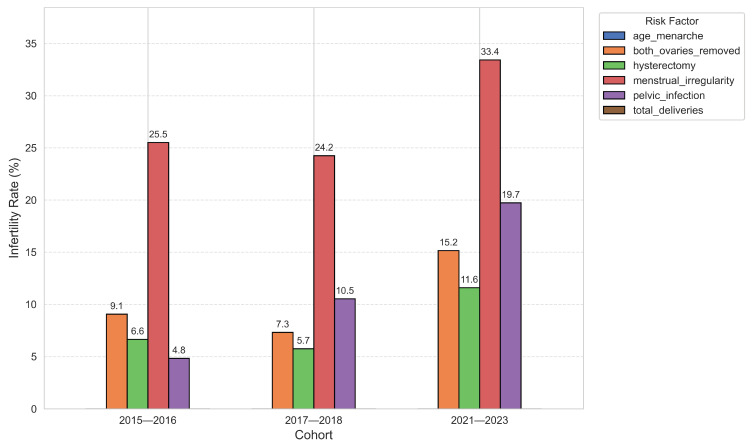
Infertility rate by common clinical risk factors across NHANES cohorts (2015–2021).

Across all NHANES cycles (2015–2023), menstrual irregularity consistently showed the highest infertility rates, rising from 25.5% in 2015–2016 to 33.4% in 2021–2023. PID and bilateral oophorectomy demonstrated the most pronounced relative increases, nearly quadrupling and doubling, respectively, over the study period (Figure 4). Hysterectomy-related infertility rates also increased steadily, while age at menarche remained negligible across cycles. Total deliveries showed persistently lower infertility rates, supporting its inverse association with infertility risk. These trends underscore a post-pandemic amplification of infertility linked to menstrual disorders, PID, and surgical reproductive history, highlighting priority targets for intervention.

### 3.5. Multivariate Analysis of Infertility Predictors

**Figure 5:**
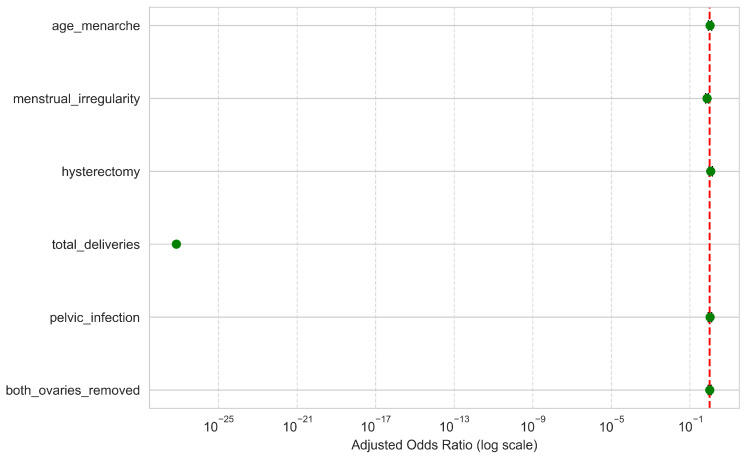
Forest plot of Adjusted Odds Ratios (Adjusted OR) for infertility predictors.

The multivariate LR model, adjusted for all included predictors (Table 2, Figure 5), revealed substantial heterogeneity in the strength and direction of associations with infertility. Total number of deliveries demonstrated a markedly strong inverse association with infertility, with an adjusted Odds Ratio (Adjusted OR) close to zero, indicating that women with prior childbirth history had significantly lower odds of reporting infertility.

Menstrual irregularity, hysterectomy, PID, both ovaries removed, and age at menarche all exhibited positive associations with infertility, though their effect sizes were modest relative to total deliveries. Among these, menstrual irregularity remained the strongest positive predictor (Adjusted OR = 0.55, 95% CI 0.40–0.77, *p* < 0.001), followed by hysterectomy and PID, reflecting their established clinical relevance in reproductive impairment. Bilateral removal of ovaries also conferred elevated infertility odds (Adjusted OR = 1.02), consistent with the irreversible loss of ovarian function, while age at menarche showed no significant association.

Overall, these findings confirm that reproductive surgical history and menstrual disorders are key independent risk factors for infertility, whereas prior childbirth exerts a strong protective influence. The stark magnitude of the delivery variable suggests that reproductive history should be carefully considered in both risk stratification and causal inference frameworks.

### 3.6. Model Performance Comparison

**Figure 6:**
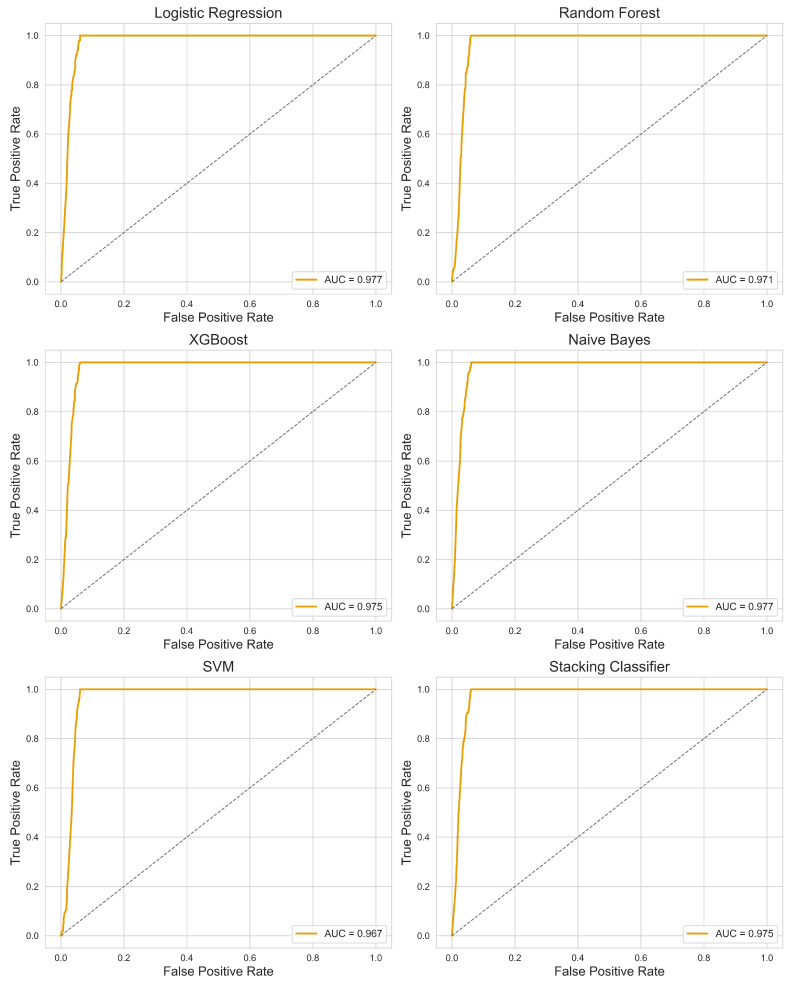
ROC curves with AUC scores for all classification models. Each subplot displays the ROC curve for one classification model evaluated on the test dataset, with the Area Under the Curve (AUC) indicated in the legend. The orange line represents the model’s discriminative performance, while the dashed diagonal gray line represents the performance of a random classifier. The ROC curve illustrates the trade-off between sensitivity (true positive rate) and 1-specificity (false positive rate) across different classification thresholds. Higher AUC values indicate better model performance.

**Figure 7:**
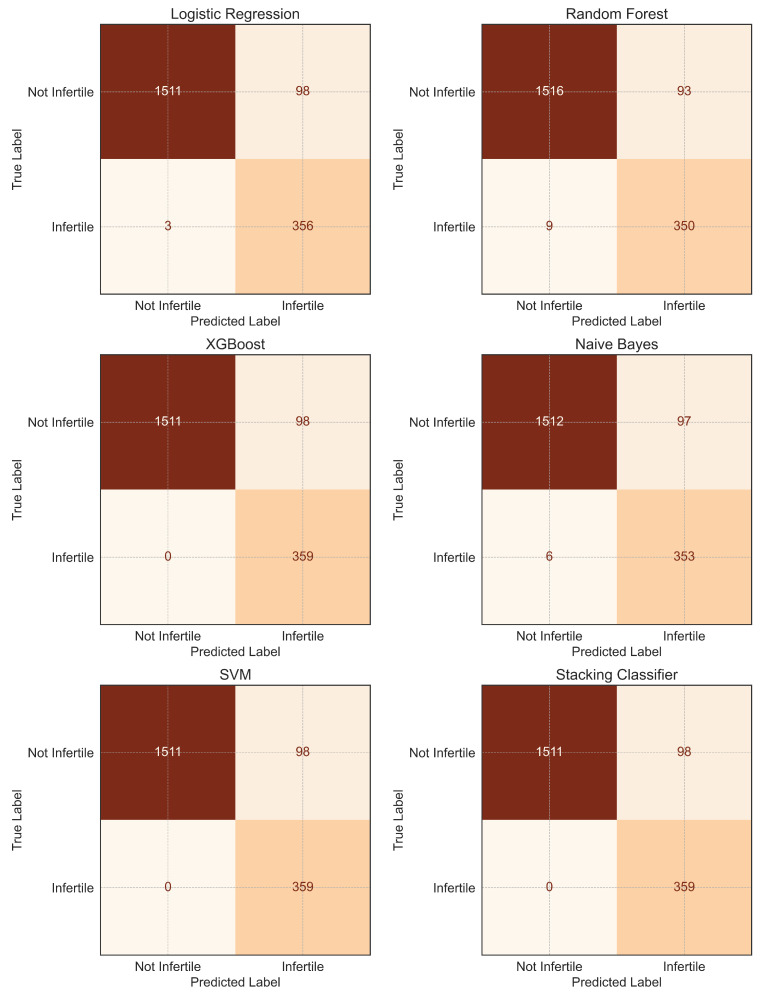
Confusion matrices showing prediction outcomes for all classification models. Each confusion matrix summarizes the classification results on the test dataset, showing the counts of true positives, true negatives, false positives, and false negatives. The x-axis indicates predicted class labels, and the y-axis indicates actual class labels, with “Not Infertile” and “Infertile” representing the two outcome categories. Color intensity reflects the magnitude of the cell counts, with distinct colormaps applied to each model for visual differentiation. The matrices provide insight into model-specific strengths and weaknesses in identifying positive and negative cases.

The predictive performance of six ML models for infertility classification was evaluated using accuracy, precision, recall, F1-score, and specificity (Table 3), with further assessment via ROC curve analysis (Figure 6) and confusion matrices (Figure 7). The dataset with selected features was split into training (70%) and testing (30%) sets. All models demonstrated high discriminative ability, with AUC values ranging from 0.967 (SVM) to 0.977 (LR and NB).

LR achieved the highest recall (0.992) alongside NB (0.983) and XGBoost/SVM/Stacking Classifier (1.000), indicating strong sensitivity in identifying infertility cases. Precision values were comparable across models (0.784–0.790), suggesting consistent performance in minimizing false positives. XGBoost, SVM, and the Stacking Classifier attained the highest F1-score (0.880), reflecting an optimal balance between precision and recall. RF showed slightly lower recall (0.975) compared to the top-performing models but maintained competitive accuracy (0.948) and specificity (0.942).

The confusion matrices revealed that most models misclassified fewer than 10 infertility cases, with LR, XGBoost, SVM, and the Stacking Classifier demonstrating perfect classification for infertile women in the test set. ROC curves further confirmed robust model performance, with all curves positioned well above the diagonal reference line, highlighting their strong predictive capability.

Optimized hyperparameters for each algorithm, determined via GridSearchCV, are presented in Table 4. For LR, the optimal configuration included an L2 penalty with the lbfgs solver and a regularization parameter C = 1. RF achieved best results with 200 estimators, a maximum depth of 5, and a minimum sample split of 2. XGBoost performed optimally with a learning rate of 0.01, maximum depth of 3, 200 estimators, and a subsample ratio of 0.8. NB required minimal tuning, with var_smoothing set to 0. For SVM, a linear kernel with C = 0.1 and gamma = scale provided the best performance. The Stacking Classifier, integrating XGBoost, RF, LR, and SVM, achieved identical top-tier results to XGBoost and SVM, reinforcing the advantage of ensemble-based approaches in infertility prediction.

## 4. Discussion

This study presents a comprehensive cross-cohort analysis of female infertility trends and associated predictors using NHANES data from 2015 to 2023 [17]. By integrating descriptive epidemiology with machine learning approaches, the analysis offers novel insights into shifting infertility patterns, particularly in the wake of the COVID-19 pandemic.

One of the most compelling findings is the marked increase in self-reported infertility in the 2021–2023 NHANES cycle, which rose from 14.8% in 2017 to 27.8%. This doubling of prevalence suggests significant post-pandemic disruptions to reproductive health. These disruptions may stem from delays in fertility-related healthcare access, heightened psychosocial stress, and broader changes in reproductive decision-making during the pandemic. Prior studies support this interpretation, highlighting reduced availability of gynecological care and postponed family planning services during COVID-19 lockdowns [8,19,20,21]. Additionally, pandemic-associated lifestyle changes and metabolic shifts may have indirectly impacted fertility outcomes [22,23]. Importantly, external national surveillance data corroborate these findings: U.S. vital statistics reported a historic decline in the general fertility rate (GFR) in 2020, dropping to 55.8 births per 1000 women aged 15, 44 years, a 4% decrease from 2019 and the sharpest single-year decline in nearly five decades [24]. Although a modest rebound occurred in 2021, live births remained below pre-pandemic levels, consistent with the elevated infertility burden observed in our analysis.

The discrepancy between the nearly 28% prevalence of self-reported infertility observed in NHANES 2021–2023 and the widely cited global estimate of 10–15% requires careful interpretation. First, our analysis relied on a self-reported infertility measure (‘attempted pregnancy for ≥12 months without success’), which may capture both biological infertility and delayed conception in women with reduced or disrupted healthcare access during the pandemic. Second, infertility prevalence in population surveys does not directly equate to live birth rates. The 4% decline in U.S. births in 2020 reflects aggregate demographic and social dynamics, whereas the NHANES-based measure reflects individual reproductive experiences and attempts to conceive. Together, these differences highlight that infertility prevalence and birth rate trends, though related, are not directly comparable and may diverge during periods of healthcare disruption and social change.

Beyond overall prevalence trends, the feature importance analysis revealed that total number of deliveries was the most dominant factor in infertility classification, with a markedly larger LR coefficient than all other predictors combined. This strong inverse relationship likely reflects the inherent definition of infertility (inability to conceive after prior attempts) and underscores the central role of reproductive history in risk assessment. Menstrual irregularity emerged as the most influential positive predictor, aligning with its established role in ovulatory dysfunction and conditions such as PCOS [25,26,27,28]. Other variables, including hysterectomy, PID, bilateral oophorectomy, and age at menarche, contributed more modestly to classification performance. This relatively weak association may stem from the survey’s reliance on self-reported infertility, which depends on whether participants actively attempted pregnancy in the past year. For example, women who have undergone hysterectomy or bilateral oophorectomy are physiologically unable to conceive, but would not report infertility if they did not attempt pregnancy, thereby attenuating observed associations.

Cohort-specific analyses further indicated that menstrual irregularity, PID, and surgical reproductive history (especially bilateral oophorectomy) have become increasingly associated with infertility in the post-pandemic period. The quadrupling of infertility rates among women with a history of PID and the doubling of rates among those with bilateral oophorectomy suggest that both infectious and surgical factors may have had amplified reproductive consequences in recent years. These trends highlight potential targets for public health interventions, such as sexually transmitted infections prevention and early gynecological care [29,30].

The adjusted multivariate LR model (Table 2, Figure 5) demonstrated notable variation in the magnitude and direction of associations with infertility. Prior childbirth emerged as a dominant protective factor, with an adjusted OR approaching zero, underscoring the substantially lower likelihood of infertility among women with previous deliveries. In contrast, menstrual irregularity, hysterectomy, PID, and bilateral oophorectomy each showed positive associations with infertility, although their effect sizes were comparatively modest. Menstrual irregularity was the strongest positive predictor (Adjusted OR = 0.55, 95% CI 0.40, 0.77, *p* < 0.001), aligning with its well-documented role in impaired fecundity. Hysterectomy and PID also contributed meaningfully, consistent with their established pathological impact on reproductive capacity. Bilateral oophorectomy was associated with higher odds of infertility (Adjusted OR = 1.02), reflecting the irreversible cessation of ovarian function, while age at menarche showed no significant effect. Collectively, these findings reinforce the importance of reproductive surgical history and menstrual disorders in infertility risk profiling, while highlighting that a history of childbirth is a potent protective factor that should be incorporated into both predictive modeling and etiologic interpretations.

ML model evaluation demonstrated uniformly high discriminative ability across all six algorithms, with AUC values exceeding 0.96 and several models, including LR, NB, XGBoost, SVM, and the Stacking Classifier, achieving near-perfect recall for infertility cases. The comparable performance of interpretable models LR and advanced ensemble approaches reinforces the utility of both strategies: simpler models for clinical interpretability and complex ensembles for maximizing predictive stability across heterogeneous datasets. The success of the Stacking Classifier, which integrated multiple base learners, supports the growing evidence that ensemble methods can optimize predictive robustness in epidemiological applications [13,15,31]. Our findings are consistent with prior work in reproductive health ML, where stacking approaches coupled with oversampling techniques significantly improved predictive performance in PCOS classification [18].

The consistency of model performance and the stability of key predictor effects validate the robustness of this approach, despite limitations. Specifically, infertility was assessed via self-report, which introduces potential recall bias. Moreover, NHANES’s cross-sectional structure precludes causality inference. Critically, the analysis excluded well-established confounders such as BMI, smoking, socioeconomic status, and age due to data harmonization constraints across cycles. This restricts the comprehensiveness of the model and may underestimate the impact of lifestyle or demographic variables. Additionally, the absence of a separate dataset for the 2019–2021 period due to COVID-19-related disruptions limited the ability to directly evaluate infertility trends during this intermediate period, potentially reducing the temporal resolution of our findings.

Nevertheless, this study provides important contributions to the field of reproductive epidemiology. It highlights post-pandemic changes in infertility burden, validates the utility of minimal-feature models for risk prediction, and emphasizes the need for targeted surveillance of menstrual and infectious disorders in reproductive health. Future research should expand variable inclusion, adopt longitudinal designs, and explore intervention effectiveness to improve fertility outcomes in vulnerable populations.

## 5. Conclusions

This study provides timely and comprehensive insights into the evolving landscape of female infertility in the United States, leveraging NHANES data across three cohorts (2015–2023). A striking rise in self-reported infertility was observed in the most recent 2021–2023 cycle, highlighting potential post-pandemic disruptions in reproductive health, including delays in care, altered lifestyle behaviors, and increased stress levels. Among the clinical predictors evaluated, reproductive history, particularly prior childbirth, emerged as the strongest protective factor against infertility. In contrast, menstrual irregularity showed a robust positive association with infertility, whereas PID, hysterectomy, and bilateral oophorectomy were not significantly associated after adjustment. ML analysis demonstrated excellent and consistent predictive performance across all models (AUC = 0.971–0.977), with XGBoost, SVM, and the stacking ensemble achieving perfect recall while maintaining high overall accuracy around 95%. Importantly, the strong and transparent performance of LR underscores its practical utility in clinical and public health applications, where interpretability is essential for risk communication and decision-making.

Despite limitations, including the exclusion of age, BMI, and socioeconomic status, and the cross-sectional nature of the data, this study underscores the need for enhanced reproductive health surveillance and early risk identification strategies. Future research should integrate broader behavioral, hormonal, and demographic variables and adopt longitudinal designs to refine infertility prediction models and inform targeted interventions. 

## Figures and Tables

**Table 1:** Descriptive statistics of the study population based on common variables across NHANES cohorts (2015–2023).

Variable	2015–2016	2017–2018	2021–2023	Total (N = 6560)	*p*-Value
Sample Size (n)	2534	2483	1543	6560	
Age Menarche (years)	12.7 ± 1.8	12.7 ± 1.8	12.3 ± 1.7	12.6 ± 1.8	p<0.001
Total Deliveries	2.4 ± 1.9	2.4 ± 4.0	1.5 ± 1.4	2.2 ± 2.9	p<0.001
Menstrual Irregularity (%)					
Yes	1168 (46.09%)	1064 (42.85%)	1017 (65.91%)	3249 (49.53%)	p=1.000
No	1366 (53.90%)	1419 (57.15%)	415 (34.09%)	3311 (50.47%)	
Hysterectomy (%)					
Yes	557 (21.98%)	557 (22.43%)	181 (11.73%)	1295 (19.74%)	p=0.348
No	1977 (78.02%)	1926 (77.57%)	1362 (88.27%)	5265 (80.26%)	
Pelvic Infection (PID) (%)					
Yes	83 (3.28%)	114 (4.59%)	76 (4.93%)	273 (4.16%)	p<0.001
No	2451 (96.72%)	2369 (95.41%)	1467 (95.07%)	6287 (95.84%)	
Ever Pregnant (%)					
Yes	2134 (84.21%)	2115 (85.18%)	1114 (72.20%)	5263 (80.23%)	p<0.001
No	400 (15.79%)	368 (14.82%)	429 (27.80%)	1197 (19.77%)	
Both Ovaries Removed (%)					
Yes	287 (11.33%)	287 (11.59%)	66 (4.28%)	640 (9.76%)	p=0.070
No	2247 (88.67%)	2196 (88.44%)	1477 (95.72%)	5920 (90.24%)	

The table summarizes clinical and reproductive health-related variables that are consistently available across all included NHANES cycles from 2015 to 2023. Values are presented as mean (standard deviation) for continuous variables and n (%) for categorical variables. *p*-values for continuous variables were calculated using one-way analysis of variance (ANOVA) across the three NHANES survey cycles (2015–2016, 2017–2018, 2021–2023). *p*-values for categorical variables were obtained using the Chi-square test of independence. Bold indicates statistical significance (p<0.05).

**Table 2:** Multivariate Logistic Regression (LR) for infertility risk: Adjusted Odds Ratios (OR), 95% confidence intervals (CI), and *p*-values for predictors.

Variable	Adjusted OR	95% CI	*p*-Value
Age menarche	1.00	1.0–1.0	0.5365
Menstrual irregularity	0.55	0.40–0.77	0.0005 *
Hysterectomy	1.36	0.88–2.09	0.1683
Total deliveries	0.00	0.0–inf	0.9919
Pelvic infection	1.05	0.87–1.28	0.6002
Both ovaries removed	1.02	0.82–1.28	0.8303

OR = Odds Ratio; CI = Confidence Interval. Adjusted for all variables listed. * *p*-value < 0.05 considered statistically significant.

**Table 3:** Performance metrics of six Machine Learning (ML) models for infertility prediction.

Model	Accuracy	Precision	Recall	F1-Score	Specificity
Logistic Regression	0.949	0.784	0.992	0.876	0.939
Random Forest	0.948	0.790	0.975	0.873	0.942
XGBoost	0.950	0.786	1.000	0.880	0.939
Naive Bayes	0.948	0.784	0.983	0.873	0.940
SVM	0.950	0.786	1.000	0.880	0.939
Ensemble (Stacking Classifier)	0.950	0.786	1.000	0.880	0.939
Base Layer: XGBoost + Random Forest					
+ Logistic Regression + SVM					
Meta-Layer: Logistic Regression					

Note: Accuracy = proportion of all correct predictions; Precision = proportion of predicted infertile cases that are true infertile; Recall (Sensitivity) = proportion of actual infertile cases correctly identified; F1-score = harmonic mean of Precision and Recall; Specificity = proportion of fertile cases correctly identified.

**Table 4:** Optimized hyperparameters for Machine Learning (ML) models in fertility classification.

Model	Parameter	Value
Logistic Regression	C	1
	penalty	l2
	solver	lbfgs
	max_depth	5
	min_samples_split	2
Random Forest	n_estimators	200
XGBoost	colsample_bytree	1
	learning_rate	0.01
	max_depth	3
	n_estimators	200
	subsample	0.8
Naive Bayes	var_smoothing	0
SVM	C	0.1
	gamma	scale
	kernel	linear

Note: This table lists the optimal hyperparameter values selected for each machine learning model after grid search and cross-validation, used in the fertility classification study. Parameters follow the scikit-learn pipeline naming convention.

## Data Availability

Publicly available datasets were analyzed in this study. These data can be accessed through the National Health and Nutrition Examination Survey (NHANES) portal maintained by the U.S. Centers for Disease Control and Prevention (CDC) at https://www.cdc.gov/nchs/nhanes/index.html, accessed on 3 July 2025.

## Abbreviations

The following abbreviations are used in this manuscript:

NHANES	National Health and Nutrition Examination Survey
PCOS	Polycystic Ovary Syndrome
PID	Pelvic Inflammatory Disease
ML	Machine Learning
LR	Logistic Regression
RF	Random Forest
XGBoost	Extreme Gradient Boosting
NB	Naïve Bayes
SVM	Support Vector Machine
AUC	Area Under the Receiver Operating Characteristic Curve
ROC	Receiver Operating Characteristic
OR	Odds Ratios
CIs	Confidence Intervals
CDC	Centers for Disease Control and Prevention
